# Nearly Panoramic Neuromorphic Vision with Transparent Photosynapses

**DOI:** 10.1002/advs.202303944

**Published:** 2023-08-27

**Authors:** Xuemei Dong, Chen Chen, Keyuan Pan, Yinxiang Li, Zicheng Zhang, Zixi He, Bin Liu, Zhe Zhou, Yueyue Wu, Dengfeng Zhang, Hongchao Sun, Xinkai Qian, Min Xu, Wei Huang, Juqing Liu

**Affiliations:** ^1^ Key Laboratory of Flexible Electronics (KLOFE) and Institute of Advanced Materials (IAM) Nanjing Tech University (Nanjing Tech) 30 South Puzhu Road Nanjing 211816 China; ^2^ Frontiers Science Center for Flexible Electronics Xi'an Institute of Flexible Electronics (IFE) and Xi'an Institute of Biomedical Materials & Engineering Northwestern Polytechnical University Xi'an 710072 China

**Keywords:** dual‐side perception, motion detection, nearly panoramic neuromorphic vision, transparent photosynapse, 2D materials

## Abstract

Neuromorphic vision based on photonic synapses has the ability to mimic sensitivity, adaptivity, and sophistication of bio‐visual systems. Significant advances in artificial photosynapses are achieved recently. However, conventional photosyanptic devices normally employ opaque metal conductors and vertical device configuration, performing a limited hemispherical field of view. Here, a transparent planar photonic synapse (TPPS) is presented that offers dual‐side photosensitive capability for nearly panoramic neuromorphic vision. The TPPS consisting of all two dimensional (2D) carbon‐based derivatives exhibits ultra‐broadband photodetecting (365–970 nm) and ≈360° omnidirectional viewing angle. With its intrinsic persistent photoconductivity effect, the detector possesses bio‐synaptic behaviors such as short/long‐term memory, experience learning, light adaptation, and a 171% pair‐pulse‐facilitation index, enabling the synapse array to achieve image recognition enhancement (92%) and moving object detection.

## Graphical abstract

A transparent planar photonic synapse (TPPS) is presented that offers dual‐side photosensitive capability for ≈360° neuromorphic visual perception and recognition of moving objects. The TPPS integrates an organic heterobilayer and carbon electrodes on a flat quartz substrate, exhibiting ultraviolet‐visible‐near‐infrared‐wavelength perception (365–970 nm) and various synaptic plasticity ascribed to persistent photoconductivity.

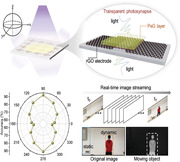

## 1 Introduction

Inspired by the biological visual systems with powerful optical sensing and processing abilities, artificial neuromorphic vision is universally recognized as an indispensable photosensing unit that can detect, classify, recognize, understand, and track multiple targets in the complex scenes.^[^ ^1^, ^2^, ^3^ ^]^ Photosynapses are promising candidates for future artificial vision due to their similar information sensing mode to neural signals, integrating perception and synaptic behaviors simultaneously.^[^ ^4^ ^]^ By converting optical information into electrical signals, photosynapse can perform photosensing, memorizing, and processing in parallel, achieving device‐level emulation of the retina and performing image preprocess such as noise reduction and contrast enhancement.^[^ ^5^, ^6^, ^7^ ^]^ To emulate the human visual system, various retinomorphic synapses using photosensitive materials such as 2D MoS_2_,^[^ ^8^, ^9^ ^]^ perovskite nanocrystals,^[^ ^10^, ^11^, ^12^ ^]^ polymer electrets,^[^ ^13^ ^]^ and organic frameworks,^[^ ^14^ ^]^ have been developed successfully, enabling target color feature extraction and selective recognition of color‐mixed pattern.^[^ ^15^, ^16^ ^]^ Apart from static images, bio‐retinas with temporal differentiation functions also accelerate the perception of moving objects. Note that most reported neuromorphic visual systems have limited maximum field of view (FoV) due to opaque components and vertical architecture,^[^ ^17^, ^18^, ^19^ ^]^ restricting them to hemispherical FoV (≈180°) with large blind area and inferior motion acquisition. Thus, developing neuromorphic devices with broadband response and omnidirectional FoV is of great significance for efficient and smart artificial visual systems.

Herein, we report a transparent planar photonic synapse (TPPS) that achieves nearly panoramic retina plausibility through the combination of organic bilayer and carbon conductor. The resulting TPPS exhibits ultraviolet‐visible‐near‐infrared‐wavelength perception and a virtually ultrawide FoV covering the whole 3D space. A variety of synaptic plasticity was achieved in this photonic‐triggered device ascribed to charge accumulation and diffusion. Finally, we designed a simulated retina system where the TPPS severs as the basic framework, demonstrating successful perception and improved recognition of motion targets by the synergy of interframe differential computation and diverse‐angle sights focus. Our work presents the new inspiration for constructing efficient and compact artificial vision using transparent photosynapses toward panoramic vision and moving target tracking.

## 2 Results and Discussion

### 2.1 Transparent Planar Photonic Synapse (TPPS) Design and Operation Principle

**Figure 1:**
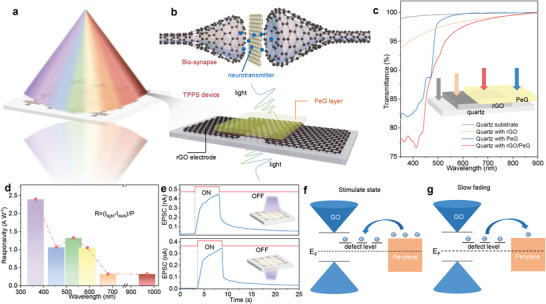
Design and operation principle of TPPS device. a) The diagram of the prepared TPPS array with dual‐side photoreception. The right side is a single device structure of PeG heterobilayer with rGO planar electrodes. b) The diagram of a biological synapse, optical spike serves as a presynaptic input spike, channel current as stimulated postsynaptic current. The charge transfer at the heterojunction interface is analogous to the release of neurotransmitter in biosynapse. c) UV–vis transmission spectrum of electrode, PeG and TPPS device. d) The wide light responsivity (ultraviolet, visible and near‐infrared) of device. e) The excitatory postsynaptic current of synapse is induced by a 365 nm light pulse with 1.44 µW mm^−2^ from upper or bottom sides. f,g) Energy band diagram of PeG with and without light stimuli, respectively.

**Figure** **1**a and Figure S1 (Supporting Information) schematically depict the TPPS based on the 2D perylene/graphene oxide (PeG) heterostructure, which closely resembles biological synapses. In biological visual perception and memory processes (Figure 1b), the presynaptic terminal receives an external light stimulus and induces an action potential, causing neurotransmitters to move from the presynaptic membrane to the postsynaptic membrane, and altering the postsynaptic current (PSC). In our TPPS device, the reduced graphene oxide (rGO) electrodes serve as presynaptic and postsynaptic electrodes, while the external light stimulus and heterojunction current correspond to presynaptic incoming signal and postsynaptic current in biosynapses. When a presynaptic light stimulus is removed, the postsynaptic current gradually returns to its original state, essential for storing, processing, and transmitting information. The submillimeter‐scale 2D PeG heterobilayer was prepared on the water surface using a liquid surface‐assisted solution co‐assembly approach, presenting a uniform and single‐crystal feature (Figure S2, Supporting Information). Figure 1c shows a high transparency (80–90%) of fabricated synaptic device with a high‐quality transparent rGO conductive array in the visible spectrum. Atomic force microscopy (AFM), scanning electron microscopy (SEM), and energy‐dispersive X‐ray spectroscopy (EDS) element mapping of C (red) and O (green) verified the uniform bilayer formation by covering perylene crystals with GO films (Figures. S3 and S4, Supporting Information). Raman results confirmed the clear combination of characteristic peaks of perylene crystal (1293 and 1366 cm^−1^) and GO (1332 and 1583 cm^−1^) without the appearance of new peaks (Figure S5, Supporting Information). The selected area electron diffraction (SAED) patterns of the perylene crystals show bright and ordered diffraction spots of (−200), (−210), (010) and electron beam direction of [200], consistent with our previous reports^[^ ^20^ ^]^ (Figure S6, Supporting Information). Figure S7 (Supporting Information) indicates the parallel crystallographic *bc* plane to the GO layer, the perylene molecules adopt a typical herringbone arrangement with a herringbone angle of 72.5. The perylene molecules in this stacking are tilted by 49.5 degrees with respect to the crystallographic *c*‐axis, accompanying strong π–π interaction between perylene and GO, which is more favorable for the optical absorption of perylene crystals due to their uniaxial transition dipole moment being nearly perpendicular to a laser beam.

Figure 1d depicts the photon‐responsivity of the TPPS with a broadband range from ultraviolet (365 nm) to near‐infrared region (970 nm), consistent with the absorption spectrum of the 2D PeG heterobilayer in Figure S8 (Supporting Information). The TPPS exhibits an excitatory photoresponse under frontal illumination (365 nm), indicating potentiation of synaptic weight due to the light‐induced generation of hole‐electron pairs (top in Figure 1e). Impressively, its high transparency enables similar photoresponse when light is incident from the backside, highlighting its panoramic detection potential. The maximal PL intensity of the PeG is lower than that of the perylene crystal, demonstrating effective charge transfer between perylene layer and GO (Figure S9, Supporting Information). According to the UPS data, the energy levels of GO and perylene are calculated, suggesting a typical type II heterostructure that enables efficient electron–hole pair dissociation and spatial transfer under the action of excited photons (Figure S10, Supporting Information). During light stimuli, electrons generated by the electron–hole pair can easily transfer to the perylene film owing to their small energy barrier, while holes move in the opposite direction into the GO film, thereby producing the photocurrent. However, active functional groups (e.g., ─OH, ─COOH) in GO act as trapping centers and capture part of the dissociated electrons in the defect levels, resulting in charge carrier accumulation at the bilayer interface. When the light is removed, the accumulated electrons slowly diffuse back to perylene, producing a persistent photoconductivity (PPC) effect that endows the device with photoelectric plasticity (Figure 1f,g).

### 2.2 Photosynaptic Characteristics

**Figure 2:**
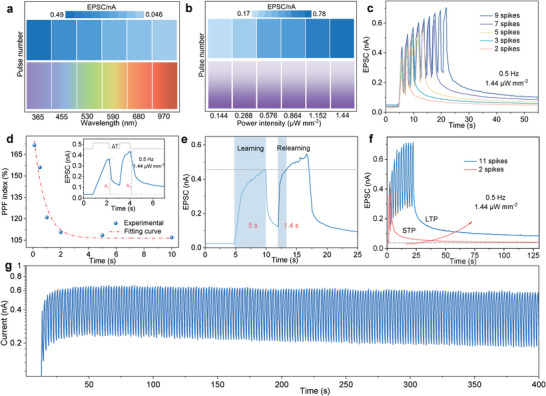
Photosynaptic characteristics. a) Influence of the wavelength on EPSC under fixed power intensity. b) Influence of the power intensity on EPSC under fixed wavelength at 365 nm. c) Influence of the pulse number on EPSC under fixed power intensity at 365 nm. d) PPF index, defined as the ratio of A_2_/A_1_, plotted as a function of the spike interval, ΔT, between two consecutive spikes. e) The measured learning‐experience behavior. f) Transfer simulation of STP and LTP under different continuous spikes. g) Light adaptability of individual photosynapse stimulated by a significantly intense UV light of 1.44 µW mm^−2^.

Understanding the response of TPPS to various visual stimuli features such as light wavelength, pulse duration, pulse number, and power intensity is crucial for imitating biological synapses. The response to continuous light pulse was plotted in **Figure** **2**a, showing peak values of excitatory post‐synaptic currents (EPSC) at diverse bands (UV–vis‐infrared) recorded with a readout bias of 0.5 V, demonstrating its wideband response. The dependence of EPSC on light power intensity (365 nm) was studied in Figure 2b, where the peak value of EPSC presented a proportional relationship with the increase in power density when keeping the number of light pulse constant. The value of EPSC also increases with the increase of light pulse duration (Figure S11, Supporting Information). Furthermore, the peak value of EPSC gradually increase as the pulse number increase (Figure 2c), and the signal induced by the latter light pulse is always stronger than the previous one during the ascent of pulse number, indicating the excellent synaptic plasticity of TPPS. Pair‐pulse‐facilitation (PPF) is a significant short‐term plasticity in biological systems that decoding temporal information in auditory or visual signals, defined as the ratio of the peak EPSC value of the second pulse to that of the first pulse.^[^ ^21^, ^22^, ^23^ ^]^ After the initial stimuli, photo‐generated electrons will gradually diffuse back to their equilibrium position on the perylene surface. By applying a following light stimulus with a smaller inter‐spike interval (ΔT), the remaining photo‐generated carriers of the initial stimulus will assist the conductance of the following one, inducing PPF behavior. We quantified this PPF by analyzing the amplitude of postsynaptic current under multiple pulses. As depicted in Figures S12 and S13 (Supporting Information), the current amplitude triggered by the second stimulus (A_2_) is significantly larger than that by the first one (A_1_). With an increment of pulse number, the amplitude amplifies gradually and reaches ≈163% for A_9_/A_1_, where the A_n_/A_1_ denotes the ratio of the nth spike current to the first one.

The spike‐time interval‐dependent plasticity (STIDP) was further explored by varying inter‐spike intervals (ΔT) from 0.1 to 10.0 s. The results show that a maximum PPF index of 171% occurs at a short interval of 0.1 s, decreasing progressively to 106% as the interval increased to 10.0 s. The attenuation curve was well fitted using a double exponential function, indicating potential for simulating PPF in biological systems (Figure 2d). Additionally, the TPPS demonstrated human‐like learning behavior with a faster relearning time compared to initial learning in accordance with the Ebbinghaus' theory.^[^ ^24^ ^]^ Under initial learning, it takes 5 s to achieve a memory level of 0.45 nA, then gradually decays after removing the stimuli. With subsequent learning, the same level only takes 1.4 s due to residual photogenerated carriers from initial stage (Figure 2e). Short‐term potentiation (STP) and long‐term potentiation (LTP), along with their transition are crucial for the psychological memory and forgetting models of biological brain.^[^ ^25^ ^]^ The conversion from STP to LTP was imitated by stimulating synapse with light pulses of different amplitudes at the same frequency (Figure 2f). The decay time was significantly extended to 130 s as the light pulses increased from 2 to 11, demonstrating a pulse number modulated transition. In response to strong light stimuli, biological iris can modulate luminous flux into eye by changing pupil size to prevent retinal damage.^[^ ^26^, ^27^ ^]^ For TPPS, the photocurrent reaches a saturation platform after multiple pulses, then decreases gradually during the subsequently continuous pulses and attenuated by 15% at 400 s (Figure 2g), indicating the human‐like adaptive visual feelings.

### 2.3 Nearly Panoramic Perception

**Figure 3:**
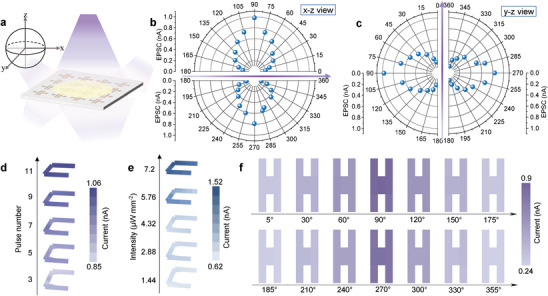
Nearly panoramic perception and recognition characterizations. a) The scheme of a TPPS array with omnidirectional optical perception. Angle‐dependent EPSC with optic pulse along b) *x*‐direction and c) *y*‐direction. d,e) Enhancement of visual learning through ultraviolet pulse with different pulse number and power density. f) The dependence of pixel current on the angle of incident light.

The TPPS's potential for constructing panoramic artificial vision was evaluated by investigating its optical uniformity and photosensitivity at different angles. As demonstrated in **Figure** **3**a, the synapse array on the quartz substrate exhibits a photoresponse at almost 0–360° along *x* direction and *y* direction due to its high transparency. The EPSC intensity dependence on azimuthal angle of a single synapse was mapped out in Figure 3b,c along *x*‐direction and *y* directions, respectively. By employing a UV‐light pulse (365 nm) with a fixed power intensity of 1.44 µW mm^−2^ and a voltage of 0.5 V, the azimuth angle of the incident light was regulated through rotating the focusing optical device, thereby affecting the light power intensity and observing the evolution of EPSC. The maximum EPSC value of 0.98 and 0.97 nA were obtained when the incident light was positioned at 90° under frontal illumination for both *x*‐ and *y*‐direction, which decayed progressively as the incidence angle decreased. When illuminated from the backside, the EPSC exhibited a similar oriented response with a maximum value of 0.79 and 0.83 nA at 270° of *x*‐ and *y‐* direction, respectively. Such azimuth‐sensitive property suggests that the TPPS has great potential for wide‐FoV detection and acquisition, making it favorable for industrial robot applications such as panoramic motion detection and obstacle avoidance.^[^ ^10^, ^28^ ^]^ To simulate the process of visual learning, we constructed a 3 × 7 pixel array and mapped changes in synaptic weight value to the EPSC, represented by grayscale values in the following image. Using ultra‐weak pulsed light (365 nm) and the letter “C” (Carbon) as the signal input, we trained the system with 3 to 11 pulses, resulting in enhanced clarity and improved resemblance to the input letters, as shown in Figure 3d. When subjected to a fixed 9‐pulses irradiation, a higher light density was observed to hasten the pattern learning process (Figure 3e). In addition, the relationship between pixel current and incident light angle (9 pulses, 1.44 µW mm^−2^) was depicted in Figure 3f, demonstrating that a more perpendicular angle (i.e., higher light intensity) results in faster pattern learning.

### 2.4 Simulated Image Recognition and Motion Detection

**Figure 4:**
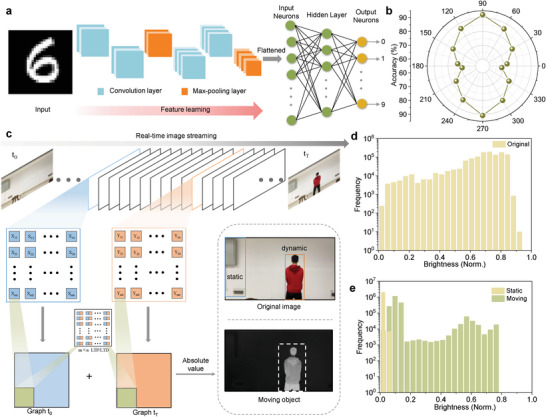
Simulated image recognition and motion detection. a) Schematic structure of TPPS‐based neural network. b) The recognition accuracy of network with different angles under 10 epochs. c) The *m×n* LTP and LTD nonlinear index are multiplied with the image pixels at different times for motion detection. d) Original pixel brightness distribution of the moving image. e) The output brightness distribution after motion detection.

To evaluate the recognition accuracy of optimized hardware array, we established an improved convolution neural network (CNN) model using the Modified National Institute of Standards and Technology (MNIST) handwriting database.^[^ ^29^, ^30^ ^]^ The model contains two feature extraction layers and a fully connected neural network (**Figure** **4**a). Using varying levels of Gaussian noise obtained from the conductivity values, we assessed the recognition accuracy of CNN model. As illustrated in our results, a maximum 92% accuracy can be obtained as incident light approached a vertical angle, simulating the recognition behaviors of biological vision (Figure 4b).

The bio‐visual system has the ability to acquire information, master new skills, and even converse. Through a combination of molecular and activity‐dependent mechanisms, neurons form intricate functional networks that enable sophisticated visual organs to identify relevant details in complex optical environments, such as motion perception.^[^ ^31^, ^32^ ^]^ In biology, the detection of moving target is achieved by the collaboration of bipolar cells and retinal neurons.^[^ ^33^ ^]^ To replicate this function, we reassemble the *m × n* pixels (*m* and *n* represent the length and width of target image, respectively) using the long‐term potentiation (LTP) and long‐term depression (LTD) nonlinear index^[^ ^34^, ^35^, ^36^, ^37^ ^]^ of the TPPS and then perform frame difference calculations. Among them, the nonlinear LTD index is fitted by the inhibition of post‐synaptic potential under the action of post light pulse voltage (1 V with 10 Hz, Figures S14 and S15, Supporting Information). The resulting motion process can be viewed as a stream of images captured at different times from t_0_ to t_T_, collectively providing valuable space‐time information that is input into the network. The X_mn_ array represents image brightness distribution at t_1_ moment, while the Y_mn_ array represents the same at t_1_ + Δt moment. We then multiply the *m × n* LTP and LTD nonlinear index with image pixels at different times, subsequently summing these antagonistic results (Figure 4c) to obtain the final output. The original pixel brightness distribution of moving image is displayed in Figure 4d, and the brightness is normalized with pixel brightness ranging from 0 to 1. If there are no moving targets during the entire time period, there is no new optical signal input to the corresponding pixel module at t_0_ + ∆t, and the output brightness of all pixels will be close to zero, which is filtered out as static noise because of the similarity sign of the absolute value of the nonlinear numerical matrix of LTP/LTD being opposite. On the other hand, the presence of moving objects during ∆t will result in a dynamic information flow scene, indicative of a combined output of both static background and moving objects. After pixel‐by‐pixel multiplication and summation in static backdrop, the output brightness approaches zero, while the moving object is clarity emerged (Figure 4e) to achieve motion perception.

## 3 Conclusion

In summary, we present a transparent photosynaptic hardware for nearly panoramic neuromorphic vision. The device features an ultrathin all‐2D structure with conductive rGO and photosensitive PeG, demonstrating dual‐side retinomorphic behaviors such as broadband response, phototunable plasticity, wide field‐of‐view, and environmental self‐adaptability. The photosynapse array offers image learning and recognition capabilities, mimicking the perception of moving objects through interframe differential computation and neural network training. Our approach is effective and can be extended to other layered materials for synaptic optoelectronics, affording new frontier for smart biomimetic visual systems and neuromorphic design applications.

## 4 Experimental Section

### Materials Availability

Perylene, Toluene, and Methanol were purchased from Aladdin. Ultrapure water (18.2 MΩ·cm) was prepared using the laboratory TANKPRO ultrapure water instrument. The above raw materials were used directly without additional purification.

### Preparation of 2D PeG Heterostructure

Graphene oxide (GO) was synthesized using a modified Hummer method in this work. To prepare the GO/methanol solution (2 mg mL^−1^), the synthetic GO power was dissolved in methanol solvent, ensuring complete dissolution through ultrasonication and centrifugation. For the preparation of perylene crystals, 150 µL perylene/toluene solution (1 mg mL^−1^) was carefully dropped on the water surface (15 mL) in a weighing bottle (50 × 35 mm). To assemble the PeG heterojunction, a weighing bottle (50 × 35 mm) was used, where 150 µL perylene/toluene solution (1 mg mL^−1^) was dropped on the water surface (15 mL) with GO solution (50 µL). Subsequently, the weighing bottle was placed at room temperature, allowing for the gradual evaporation of toluene. After the solvent evaporated, the large‐area PeG heterostructure was obtained.

### PeG Heterostructure Characterizations

POM (NIKON, LV100ND), SEM (JSM‐7800F) and AFM (Park, XE‐70) were employed to characterize the morphologies and structures. The UV–vis absorption spectra were recorded on a UV‐1780 spectrophotometer. Under the 633 nm line of a He‐Ne laser, Raman spectroscopy confirm the growth of perylene on GO. The powder X‐ray diffraction (XRD) patterns were obtained via a Rigaku Smartlab X‐ray diffractometer. Covering operating 2θ angle ranges of 5 to 30 with 0.02 Å step length. The transmission electron microscopy (TEM) and selected‐area electron diffraction (SAED) were performed using a USA FEI TF20, operating at 200 kV accelerating voltage.

### Transparent Planar Photonic Synapse (TPPS) Device Fabrication

The patterned rGO electrodes were prepared from solution‐processed GO films via an oxygen‐plasma etching approach. The GO dispersion in methanol (2 mg mL^−1^) was spin‐coated on the pre‐cleaned quartz at 2000 rpm. The obtained GO layers were thermally annealed at 1000 °C to achieve high conductive rGO film. To define the contact pattern, 60 nm aluminum was thermally evaporated under vacuum onto the rGO film through a copper‐grid mask. Subsequently, the sample was exposed to oxygen plasma to etch these rGO regions uncovered by aluminum. The aluminum served here as a “sacrificial” metal, protecting the covered rGO part from ablation by plasma. The choice of aluminum was motivated by its low cost and sustainability in O_2_ plasma, together with its simple removal through a wet chemical process in an acid solution. The target rGO‐coated quartz substrate was inserted obliquely into the water solution near the backside of PeG, followed by lifting the heterostructure from solution surface. Then, the sample was put on a hot plate and dried at 50 °C under ambient conditions.

### Photosynapse Measurements

All electrical and optical properties were characterized using the Keithley 4200 SCS semiconductor parameter analyzer under environmental conditions. Measure photogenerated current by applying a bias voltage to the two terminals of the planar device through a probe. Adjust light pulses of different intensities, frequencies, and wavelengths through high‐power LED drivers (THORLABS, DC2200 Terminal) and series LED light sources (THORLABS, M365L3, M455L4, M530L4, M590L4, M625L4, M680L4, and M970L4). In the time‐current mode, by adjusting the light conditions, including the light duration, pulse frequency, radiant intensity, and pulse number, multiple control groups were established in the experiment to explore the changes in the performance of photodetectors and photosynapses. By studying the synaptic behavior of various synaptic devices to different light signals, the light response of artificial visual arrays was studied.

### Convolution Neural Network (CNN) Simulation

The hand‐written digital graph (28 × 28 pixels) in the MNIST dataset was feature extracted using convolutional kernels and activated by the RELU function. The convolutional kernels are trained based on the Keras platform. After two consecutive convolutions, the image went through a max pooling process, which was repeated twice. The output format of the pooling layer is 7 × 7 × 6, which was converted into a 294‐dimensional vector (X_0_‐X_293_) and fed into 294 input neurons. Similarly, the RELU function was activated, and the vector passed through a hidden layer with 200 neurons before finally being transformed into ten output neurons (Y_0_‐Y_9_ corresponds to the output values of the numbers 0–9 respectively) (Table S1, Supporting Information). The activation process is as follows:

(1) $$F\;\left(x\right)=\;max\left(0,x\right)$$

Compared to traditional sigmoid function, the RELU function can speed up the training process and overcome the vanishing gradient problem. Three thousand handwritten digits were selected as a test set and added Gaussian noise to the dataset in proportion to the conductivity to simulate the clarity of objects seen at different angles. Gaussian noise refers to noise whose probability density function follows a Gaussian distribution with a mean of µ and a standard deviation of σ.

(2) $$p_{\left(a<y<b\right)}=\int_{a}^{b}P\left(y\right)dy\;=\frac{1}{\sigma \sqrt{2\pi }}\;\int_{a}^{b}e^{-\frac{{\left(y-\mu \right)}^{z}}{2\sigma^{2}}}dy$$

The CNN simulation was carried out on the Crosssim platform (https://cross‐sim.sandia.gov).

The fitting of LTP/LTD are calculated according to the following functions:

(3) $$G_{LTP}=\;B\left(1-e^{\left(-\frac{P}{A}\right)}\right)+G_{min}$$

(4) $$G_{LTD}=\;B\left(1-e^{\left(\frac{P-P_{MAX}}{A}\right)}\right)+G_{max}$$

(5) $$B\;=\frac{\left(G_{max}-G_{min}\right)}{\left(1-e^{\left(-\frac{P_{max}}{A}\right)}\right)}\;$$

G_LTP_ and G_LTD_ represent the conductance of LTP and LTD, respectively. G_max_, G_min_, and P_max_ are directly extracted from experimental data, representing the maximum conductance, minimum conductance, and maximum number of pulses required to switch the device between its minimum and maximum conductance states. The fitting of LTP/LTD was implemented using MATLAB software.

## Conflict of Interest

The authors declare no conflict of interest.

## Supporting information

Supporting Information

## Data Availability

Research data are not shared.
