# Supplementary material for: Nearly Panoramic Neuromorphic Vision with Transparent Photosynapses
Source: Adv Sci (Weinh). 2023 Aug 27;10(30):2303944. doi: 10.1002/advs.202303944 (PMC10602561; doi:10.1002/advs.202303944)
Supplement: Supplementary file 1 — Supporting Information [file ADVS-10-2303944-s001.pdf]

ADVANCED SCIENCE
Open Access

Supporting Information

for *Adv. Sci.*, DOI 10.1002/advs.202303944

Nearly Panoramic Neuromorphic Vision with Transparent Photosynapses

Xuemei Dong, Chen Chen, Keyuan Pan, Yinxiang Li, Zicheng Zhang, Zixi He, Bin Liu, Zhe Zhou*, Yueyue Wu, Dengfeng Zhang, Hongchao Sun, Xinkai Qian, Min Xu, Wei Huang* and Juqing Liu**

Supporting Information

Nearly panoramic neuromorphic vision with transparent photosynapses

Xuemei Dong,[#] Chen Chen,[#] Keyuan Pan, Yinxiang Li, Zicheng Zhang, Zixi He, Bin Liu, Zhe Zhou,* Yueyue Wu, Dengfeng Zhang, Hongchao Sun, Xinkai Qian, Min Xu, Wei Huang,* Juqing Liu**

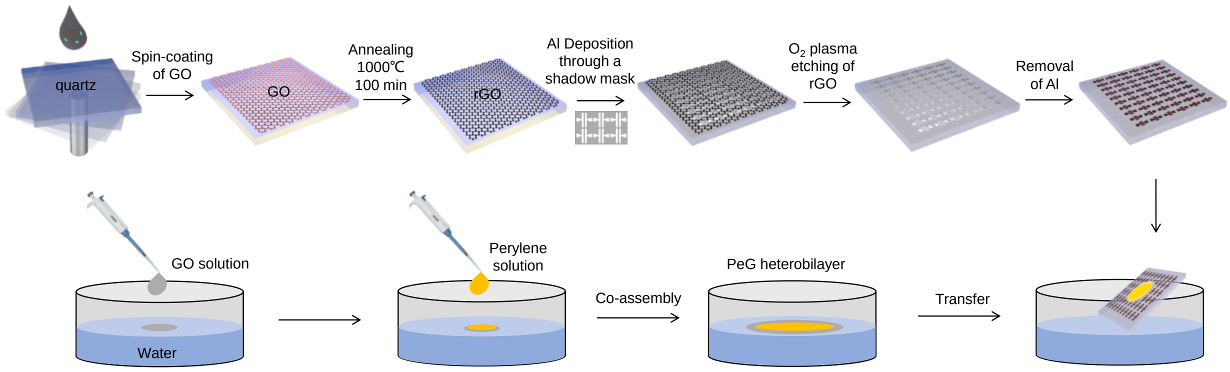

Figure S1. Schematic illustration of the approach to fabrication of the transparent planar photonic synapse device with patterned rGO electrodes.

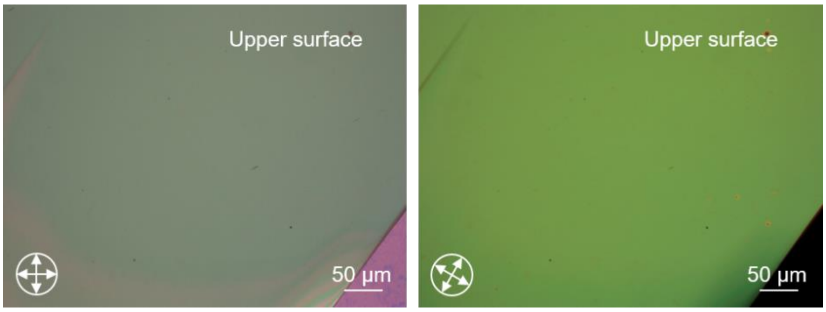

Figure S2. Polarization optical microscope images of the upper layer from the PeG heterobilayer.

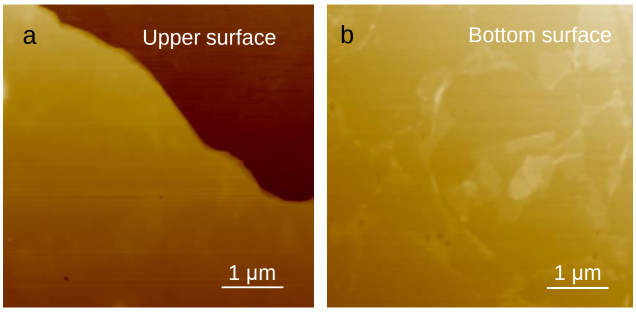

Figure S3. AFM images of **a** the upper surface and **b** the bottom surface of the PeG heterobilayer film.

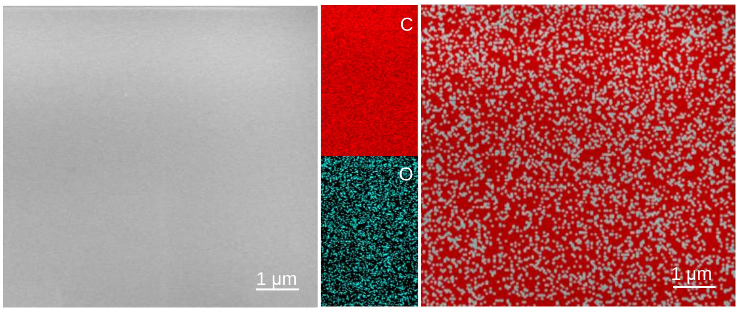

Figure S4. SEM images of PeG heterobilayer with the upper layer of GO and element mapping of C and O using EDS on SEM.

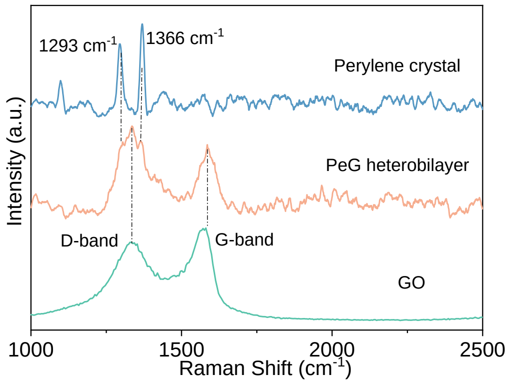

Figure S5. Raman spectra with GO, perylene crystal and PeG heterobilayer.

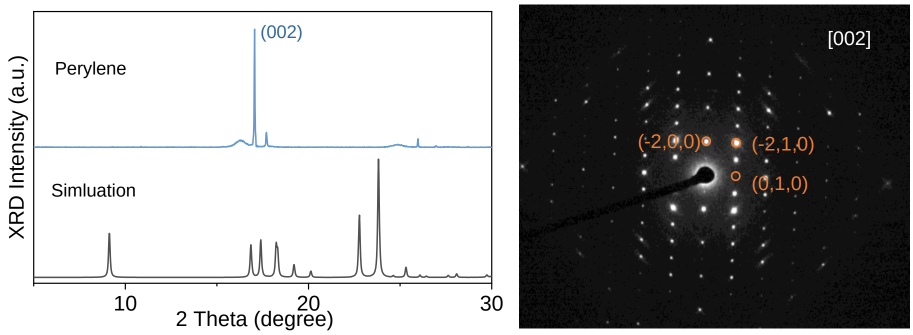

Figure S6. XRD data and SAED image of perylene crystal.

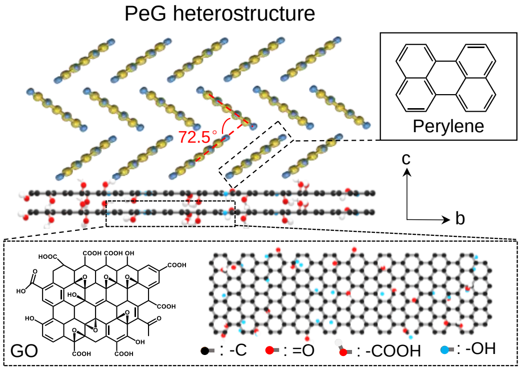

Figure S7. Ordered molecular arrangement of perylene crystal viewed from the *a* axis of the lattice and GO layer.

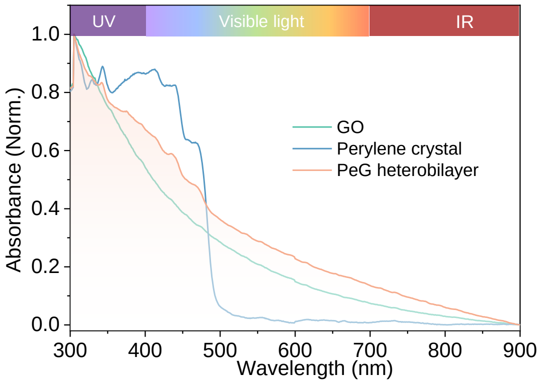

Figure S8. UV-visible to NIR absorption spectrum of GO, perylene crystal and PeG heterobilayer.

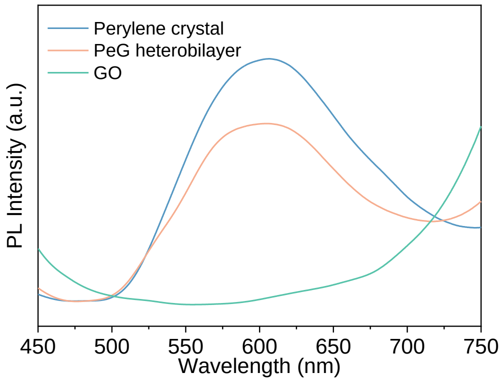

Figure S9. Steady-state PL spectrum of GO, perylene crystal, and PeG heterobilayer.

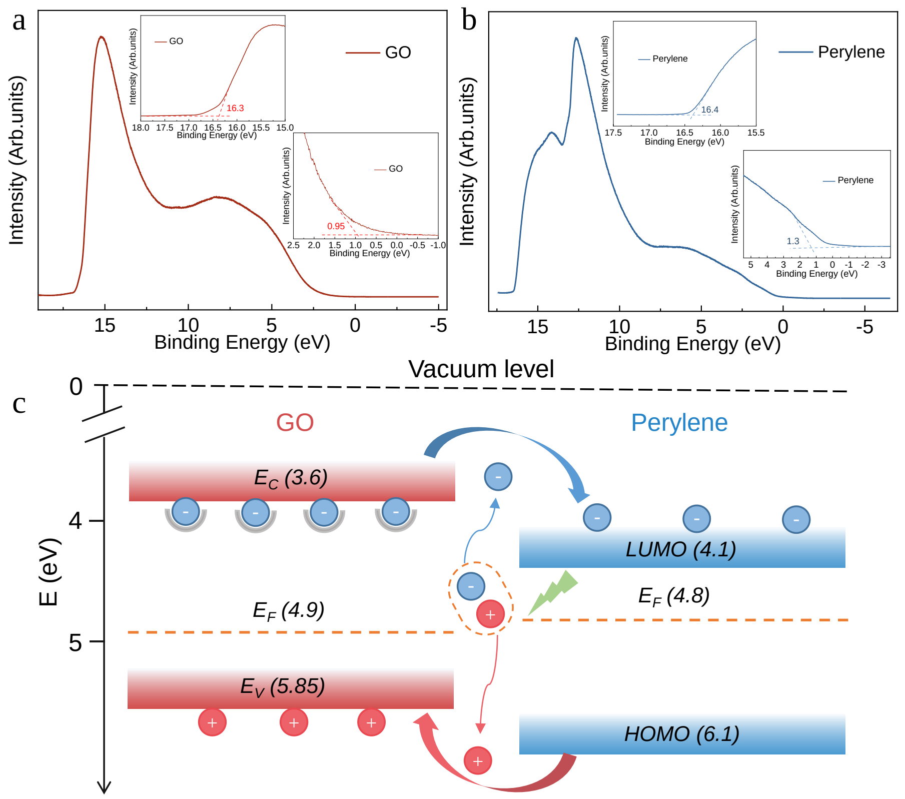

Figure S10. a-b) The UV photoelectron spectroscopy (UPS) of GO and perylene. **c**) The band structures of the PeG heterostructure.

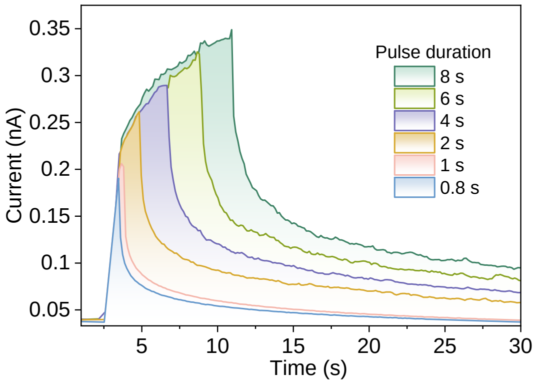

Figure S11. The EPSC change of the TPPS device as a function of the light pulse duration ranging from 800 ms to 10 s. Optical pulse: $\lambda = 365$ nm; light intensity = 1.44 μW mm^{-2}.

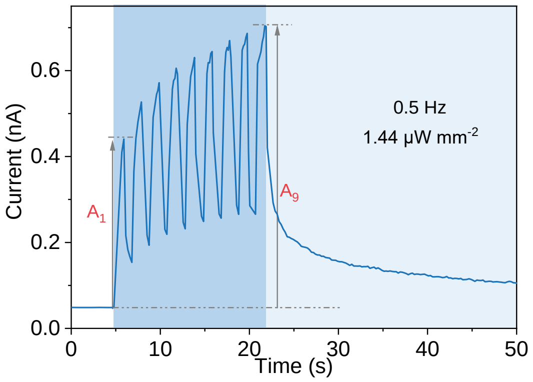

Figure S12. Measured photocurrent generated by multiple light pulses and the definition of the index (A_9/A_1).

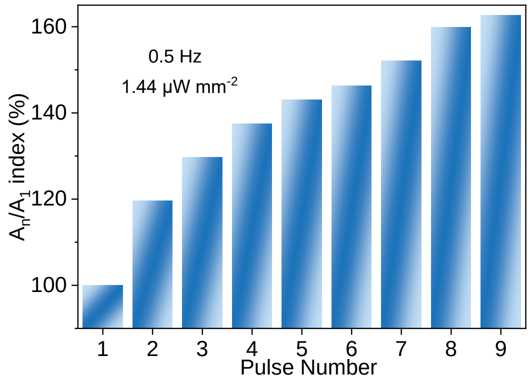

Figure S13. The evolution of the A_n/A_1 index with the pulse number.

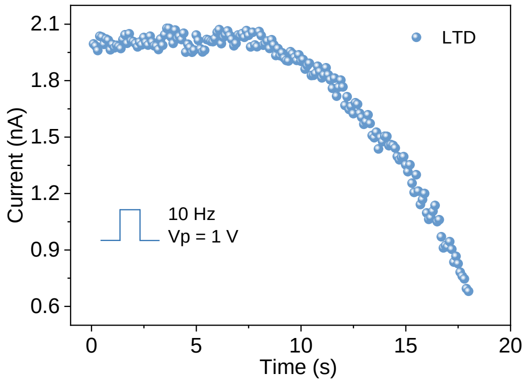

Figure S14. Inhibitory postsynaptic potential behavior under pulsed voltage (1 V 10 Hz) after illumination.

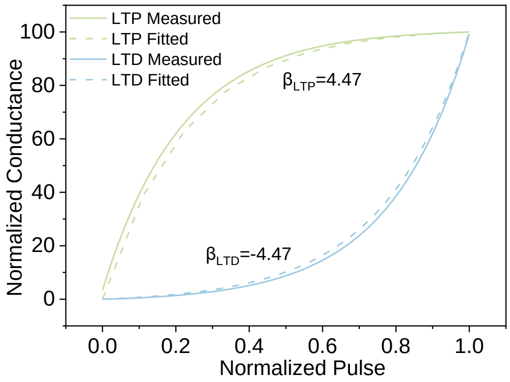

Figure S15. Nonlinearity analysis of LTP/LTD curves.

Table S1. CNN network structure.

Layer (type)	Output shape	Param
input_1 (Input Layer)	(28, 28, 1)	0
conv2d_1 (Conv2D)	(28, 28, 3)	30
re_lu_1 (ReLU)	(28, 28, 3)	0
conv2d_2 (Conv2D)	(28, 28, 3)	84
re_lu_2 (ReLU)	(28, 28, 3)	0
max_pooling2d_1 (MaxPooling2)	(14, 14, 3)	0
conv2d_3 (Conv2D)	(14, 14, 6)	168
re_lu_3 (ReLU)	(14, 14, 6)	0
conv2d_4 (Conv2D)	(14, 14, 6)	330
re_lu_4 (ReLU)	(14, 14, 6)	0
max_pooling2d_2 (MaxPooling2)	(7, 7, 6)	0
flatten_1 (Flatten)	(294)	0
dense_1 (Dense)	(200)	59000
re_lu_5 (ReLU)	(200)	0
re_lu_5 (ReLU)	(10)	2010
activation_1 (Activation)	(10)	0
